# Serum anti-NMDA-receptor antibodies and cognitive function after ischemic stroke (PROSCIS-B)

**DOI:** 10.1007/s00415-022-11203-x

**Published:** 2022-06-19

**Authors:** Pia S. Sperber, Pimrapat Gebert, Leonie H. A. Broersen, Shufan Huo, Sophie K. Piper, Bianca Teegen, Peter U. Heuschmann, Harald Prüss, Matthias Endres, Thomas G. Liman, Bob Siegerink

**Affiliations:** 1 Charité–Universitätsmedizin Berlin, Corporate Member of Freie Universität Berlin, Humboldt-Universität Zu Berlin, and Berlin Institute of Health, Center for Stroke Research Berlin (CSB), Charitéplatz 1, 10117 Berlin, Germany; 2 German Centre for Cardiovascular Research DZHK, Partner Site, Berlin, Germany; 3 German Center for Neurodegenerative Disease DZNE, Partner Site, Berlin, Germany; 4 Charité–Universitätsmedizin Berlin, Corporate Member of Freie Universität Berlin, Humboldt-Universität Zu Berlin, and Berlin Institute of Health, NeuroCure Clinical Research Center (NCRC), Berlin, Germany; 5 Charité–Universitätsmedizin Berlin, Corporate Member of Freie Universität Berlin, Humboldt-Universität Zu Berlin, and Berlin Institute of Health, Institute of Biometry and Clinical Epidemiology, Berlin, Germany; 6 Berlin Institute of Health (BIH), Charité–Universitätsmedizin Berlin and Max Delbrück Center for Molecular Medicine in the Helmholtz Association, Berlin, Germany; 7 Charité–Universitätsmedizin Berlin, Corporate Member of Freie Universität Berlin, Humboldt-Universität Zu Berlin, and Berlin Institute of Health, Department of Neurology with Experimental Neurology, Berlin, Germany; 8 Charité–Universitätsmedizin Berlin Corporate Member of Freie Universität Berlin, Humboldt-Universität Zu Berlin, and Berlin Institute of Health, Institute of Medical Informatics, Berlin, Germany; 9 Institute of Experimental Immunology, Euroimmun AG, Luebeck, Germany; 10 University of Würzburg, Institute of Clinical Epidemiology and Biometry, Würzburg, Germany; 11 University Hospital Würzburg, Clinical Trial Center Würzburg, Würzburg, Germany; 12 Leiden University Medical Center, Leiden University, Department of Clinical Epidemiology, Leiden, The Netherlands

**Keywords:** Stroke, Ischemia, Epidemiology, Antibodies, Cognitive dysfunction

## Abstract

**Objective:**

We aimed to investigate whether serum anti-N-methyl-D-aspartate-receptor GluN1 (previously NR1) antibody (NMDAR1-abs) seropositivity impacts cognitive function (CF) in the long term following ischemic stroke.

**Methods:**

Data were used from the PROSpective Cohort with Incident Stroke-Berlin. NMDAR1-abs (IgM/IgA/IgG) were measured with cell-based assays from serum obtained within 7 days after the first-ever stroke. Seropositivity was defined as titers ≥ 1:10, low titers as ≤ 1:100 and high titers as > 1:100. We assessed CF at 1, 2 and 3 years after stroke with the Telephone Interview for Cognitive Status-modified (TICS-m) and used crude and propensity score adjusted inverse probability weighted generalized linear models to estimate the impact of NMDAR1-abs serostatus on TICS-m.

**Results:**

Data on NMDAR1-abs (median day of sampling = 4[IQR = 2–5]) were available in 583/621 PROSCIS-B patients (39% female; median NIHSS = 2[IQR = 1–4]; median MMSE = 28[IQR:26–30]), of whom 76(13%) were seropositive (IgM:* n* = 48/IgA: *n* = 43/IgG: *n* = 2). Any NMDAR1-abs seropositivity had no impact on TICS-m compared to seronegative patients (βcrude = 0.69[95%CI = – 0.84 to 2.23]; βadjusted = 0.65[95%CI = – 1.00 to 2.30]). Patients with low titers scored better on TICS-m compared to seronegative patients (βcrude = 2.33[95%CI = 0.76 to 3.91]; βadjusted = 2.47[95%CI = 0.75 to 4.19]); in contrast, patients with high titers scored lower on TICS-m (βcrude =  –2.82[95%CI = – 4.90 to – 0.74], βadjusted = – 2.96[95%CI = – 5.13 to – 0.80]), compared to seronegative patients.

**Conclusion:**

In our study, NMDAR1-abs seropositivity did not affect CF over 3 years after a first mild to moderate ischemic stroke. CF differed according to NMDAR1-abs serum titer, with patients with high NMDAR1-abs titers having a less favorable cognitive outcome compared to seronegative patients.

**Supplementary Information:**

The online version contains supplementary material available at 10.1007/s00415-022-11203-x.

## Introduction and background

Cognitive impairment is frequent after stroke and up to one-third of all stroke patients develop incident post-stroke dementia.[1, 2] N-methyl-D-aspartate (NMDA) receptors are types of ionotropic glutamate receptors, sensibly regulating mechanisms of neuroplasticity, memory and cognition; however, they also play an important role in excitotoxic damage.[3] Anti-NMDA (N-methyl-D-aspartate)-receptor GluN1 (also NR1) antibodies (NMDAR1-abs) were first described in the context of a severe neuropsychiatric disease, today known as anti-NMDA-receptor encephalitis.[4] Serum NMDAR1-abs, primarily of the IgA and IgM isotypes, have been observed in about 10% of the apparently healthy and differently diseased populations.[5, 6] Some studies found associations between seropositivity and cognitive impairment.[7, 8] Previously, NMDAR1-abs seropositivity was proposed to exert beneficial effects in stroke pathology, supported by smaller infarct lesion growth in NMDAR1-abs seropositive patients in a large study of ischemic stroke patients.[9, 10] We hypothesized that NMDAR1-abs modify NMDAR function leading to altered cognitive outcome in seropositive patients following a stroke event. Therefore, we aimed to study the effects of NMDAR1-abs seropositivity on cognitive outcome in the long term after stroke in a large cohort of first-ever stroke patients.

## Materials and methods

### The PROSpective Cohort with Incident Stroke-Berlin (PROSCIS-B) study

The PROSCIS–B study (ClinicalTrials.gov identifier: NCT01363856) is a prospective observational hospital-based cohort study, which recruited patients at three tertiary university hospital stroke units of the Charité–Universitätsmedizin Berlin with first-ever stroke according to WHO criteria,[11] to study stroke secondary risks. Patients presenting with brain tumor or brain metastasis of a tumor of other origin, or patients participating in an intervention study, were excluded. Furthermore, we only included patients presenting without moderate to severe aphasia due to ethical regulations. Details on the study design have been described previously.[12, 13] For a detailed baseline characterization, an extensive clinical and technical examination was performed within 7 days after the acute event including blood sampling for laboratory measures. Magnetic resonance imaging (MRI) data were additionally collected retrospectively from clinical records and therefore did not follow standardized protocols. Patients were followed up annually by telephone interviews or postal mail contact assessing, i.a., cognitive function and functional outcome up to three years after the index event. For this investigation, only patients with mild-to-moderate ischemic stroke events (National Institutes of Health Stroke Scale [NIHSS] < 16) were included, as we counted very few cases with severe strokes (NIHSS > 15, *n* = 6).

### Assessment of anti-NMDA-receptor antibodies

Serum blood samples were obtained from patients within 7 days after stroke and stored at  – 80 °C until they were first-ever thawed for antibody measurements. NMDAR1-abs IgM, IgA and IgG were measured with cell-based assays by the Euroimmun laboratory in Luebeck, Germany. Briefly, HEK293 cells were transfected with GluN1 subunits of NMDA receptors to bind antibodies of the IgM, IgA and IgG isotype from patient serum. Fluorescein isothiocyanate anti-human IgM, IgA and IgG were secondarily administered to manually obtain staining with fluorescence microscopy. The assessors had no insight into patient data. Details on the procedure have been described elsewhere.[4, 14] Titer levels started from a dilution of 1:10, which defined seropositivity in our study. For sub-groups, we a priori defined titers of 1:10 to 1:100 as low titers and titers > 1:100 as high titers, in line with previous analyses [15]. We additionally tested sera for IgG isotype ﻿glutamic acid decarboxylase 65 kDa isoform (GAD65), gamma-aminobutyric acid B receptor (GABA-B), aquaporin 4 (AQP4), leucine-rich glioma-inactivated 1 (LGI1) and contactin-associated protein-like 2 (CASPR2) antibodies using the same CBA as described above.

### Outcome definitions

Cognitive function at baseline was measured with the Mini Mental State Examination (MMSE) and cognitive impairment at baseline was defined as MMSE < 26 [16]. For our main outcome of interest, we used the validated German version of the Telephone Interview for Cognitive Status-modified (TICS-m) to annually assess cognitive status after stroke [17]. TICS-m is a screening instrument for cognitive impairment consisting of 20 questions, the points of which add up to a maximum of 50 points total. Requested items and subscores are listed in Supplemental Methods 1. The maximal sum of points from all items scores 50. TICS-m was assessed at 1, 2 and 3 years after stroke. Patients confirmed enough time and a quiet and non-distracting environment for the time of assessment.

### Statistical methods

We calculated generalized linear models with time-specific weights (IPW GLM) to estimate the impact of NMDAR1-abs seropositivity on TICS-m over time compared to seronegative patients. We chose this statistical model because we recorded missing data in our outcome variable (TICS-m). This approach was implemented by the xtrccipw built-in command in Stata [18]. For more details on the procedure, please see Supplemental Methods 2. We included time in years on a continuous scale as time variable and the patient identifier (patient ID) to indicate dependencies of the outcomes (autocorrelation) due to repeated TICS-m measurements within one subject. The estimated effect sizes (βs) for our NMDAR1-abs exposure groups indicate the difference of TICS-m sum scores of seropositive patients compared to scores from seronegative patients over three annual measurements of follow-up. Ninety-five percent confidence intervals (95% CI) were calculated as measures of precision. We calculated a crude comparison and adjusted analyses with a propensity score as covariable, to adjust for potential confounders. Confounding variables were defined as those variables with a possible impact on NMDAR1-abs serostatus and cognitive function after stroke, selected by a causal diagram [19]. For more details on the confounder selection strategy, please see Supplemental Methods 3 and Supplemental Fig. 1. Based on the assumptions as drawn in the diagram in Supplemental Fig. 1, we ultimately considered age (continous), sex (binary), education in 2 categories (≤ 10 years of school; > 10 years of school corresponding to low-to-middle level and high level education according to the German schooling system), current smoking (yes/no), habitual alcohol consumption (yes/no) and the TOAST criteria (categories: 1. large-artery atherosclerosis, vs. 2. cardioembolism /3. small vessel occlusion /4. other cause / 5. unknown etiology) as confounders. The propensity score was then calculated with a logistic regression model and for NMDAR1-abs seropositive subgroups (i.e., high titer group and low titer group) with an ordinal logistic regression model and included into the model as covariable [20].

**Fig. 1:**
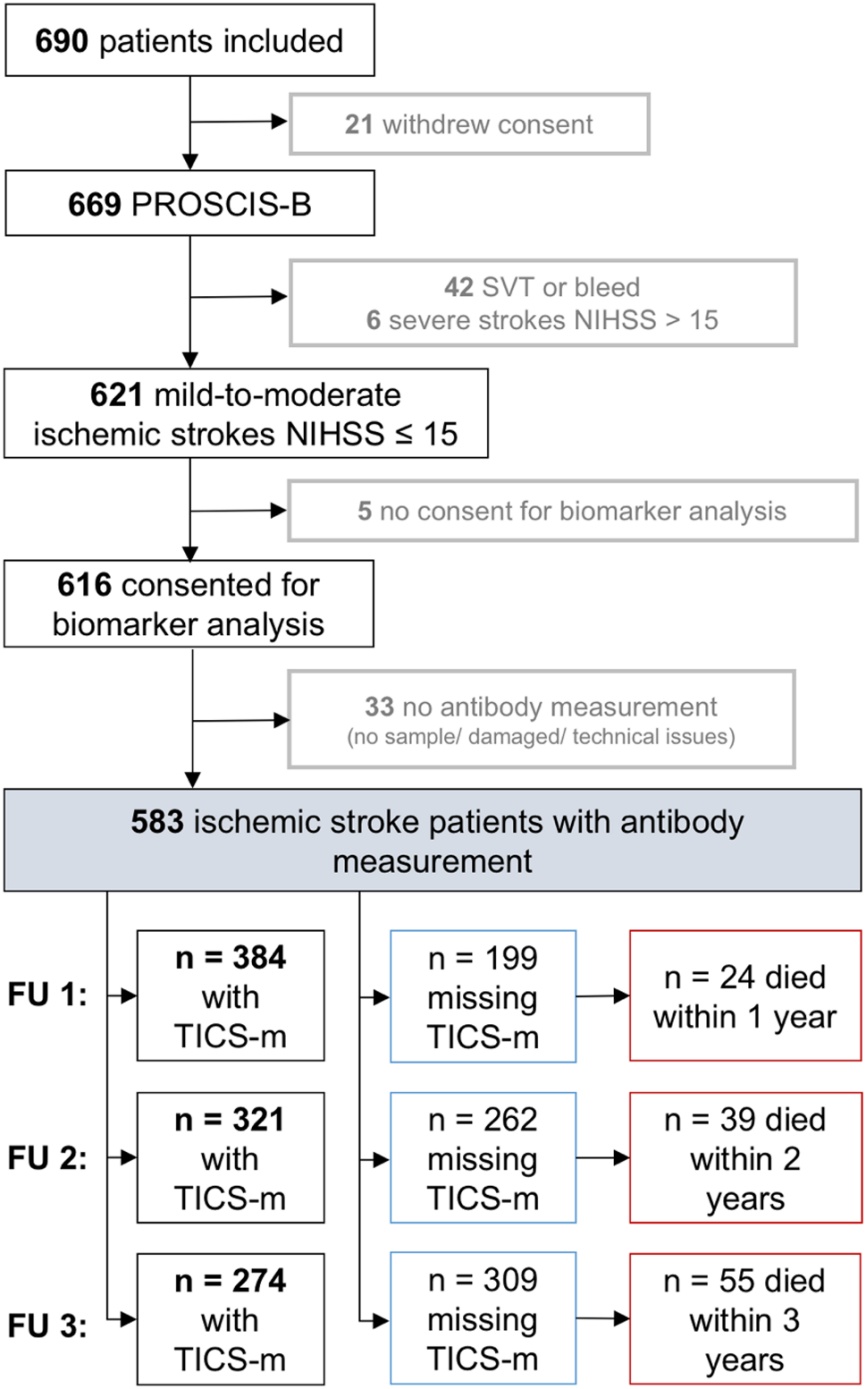
Flowchart of PROSCIS-B inclusion and exclusion and overview on follow-up data on cognitive function

### Sensitivity analyses

Firstly, we contrasted the baseline characteristics of patients who contributed at least one TICS-m score to the analyses to baseline characteristics from those patients for whom we were not able to obtain any TICS-m score at all. This approach was to explore a potential bias due to dropout indicated by baseline characteristics.

Secondly, to rule out that the effects are mainly driven by significant pre-stroke neuropsychiatric disorders, we excluded patients with an antidementia medication or antidepressant medication before stroke. A list of the Anatomic Therapeutic Chemical [ATC] codes can be found in Supplemental Methods 4, which includes: anticholinesterases, other antidementia drugs, non-selective monoamine reuptake inhibitors, selective serotonin reuptake inhibitors, non-selective monoamine oxidase inhibitors, monoamine oxidase A inhibitors or other antidepressants. After exclusion, we calculated the IPW GLMs again.

Lastly, we considered that depression is also a frequent sequela after stroke and may coincide with cognitive impairment. In an attempt to extract an isolated effect of seropositivity on cognitive function, i.e., to separate cognitive function from depression, we excluded TICS-m observations that were obtained while a patient was depressed, as defined by a score on the Center for Epidemiologic Studies Depression Scale (CES-D) questionnaire ≥ 16 [21, 22]. We calculated a best-case scenario (BC) and a worst-case scenario (WC), due to missing data in the CES-D. For more information, please see Supplemental Methods 5. We ran linear mixed models with a random effect for the patient identifier and a propensity score as covariable to adjust for confounding, similar to the main analysis, but without time-specific weights because of the active exclusion.

Data preparation was done in IBM SPSS Statistics for Windows, version 24 (IBM Corp., Armonk, N.Y., USA). Causal diagram for variable selection was drafted with DAGitty (http://www.dagitty.net/dags.html). Data visualization was conducted in R i386 3.5.1, the R foundation, with the RStudio interface using the ggplot2 package. All statistical analyses were performed using Stata version 14.2 (Stata Corp., College Station, TX, USA).

### Ethics approval

All patients or their legal guardian gave written informed consent for study participation. PROSCIS-B was approved by the local ethics committee of the Charité–Universitätsmedizin Berlin and the study was conducted in concordance to ethical principles framed by the Declaration of Helsinki.

## Data availability statement

The data and software scripts that support the findings of this study are available from the qualified principal investigator of PROSCIS-B (T.G. Liman, thomas.liman@charite.de) upon reasonable request.

## Results

### Main results

PROSCIS-B recruited patients between March 2010 and February 2013 at three campuses of the Charité–Universitätsmedizin Berlin, of whom 621 presented with mild-to-moderate ischemic stroke and were thus eligible for analyses. Of those, NMDAR1-abs measurements were conducted in 583 patient samples and the median day of blood sampling from index stroke was 4 (IQR = 3 to 5) in overall seropositive patients, 4 (IQR = 3 to 6) in patients with low titers and 4 (IQR = 3 to 5) in patients with high titers. Details on the numbers of patient inclusion and exclusion are presented in the flowchart in Fig. 1, which further provides an overview of missing TICS-m observations at each follow-up time point. Five-hundred and eighty-three patients with an antibody measurement accounted for 1749 possible follow-up assessments, from which we were able to obtain 1095 TICS-m scores. We were able to obtain at least one TICS-m score in 425 patients, rendering 158 patients who were missing all three TICS-m assessments. Fifty-five patients died, accounting for 113 (17%) of the missing observations in total.

### Baseline data

PROSCIS-B patients had a mean age of 67 years (standard deviation [SD] = 13), 39% were female and median baseline NIHSS was 2 (IQR = 1 to 4). We measured NMDAR1-abs seropositivity in 76 patients (13%) and IgM NMDAR1-abs were present in the serum of 49 patients (8%), IgA in 43 patients (7%), and IgG antibodies in 2 patients (0.3%) only. Seventeen patients (3%) presented with IgM and IgA antibodies simultaneously. No other antibody was observed, except that one patient had serum ﻿LGI1 antibodies with a low titer of 1:10. We did not observe major differences in the baseline characteristics between NMDAR1-abs seropositive and seronegative patients and also between subgroups. More seropositive patients were male and had greater MR diffusion-weighted imaging (DWI) lesion volumes, with the greatest extent in those patients with high titers (see Table 1). Group comparisons are shown in Supplemental Table 1 of the supplemental material (difference in means, logarithmic means, ranks or percentage points with 95%CI).

**Table 1:** Baseline characteristics of PROSCIS-B participants

	PROSCIS-B	Anti-NMDAR GluN1 antibody serostatus			
	Total	Seronegative	Seropositive	Titer ≤ 1:100	Titer > 1:100
PROSCIS-B participants a *n(%)*	621 (100)	507 (82)	76 (13)	55 (9)	21 (4)
Anti-NMDAR GluN1 antibodies *n(%)*					
IgM	49 (8)	–	49 (8)	34 (6)	15 (3)
IgA	43 (7)	–	43 (7)	31 (5)	12 (2)
IgG	2 (> 0)	–	2 (> 0)	2 (> 0)	0
Age (years)					
*Mean (SD)*	67 (13)	67 (13)	66 (14)	65 (14)	71 (10)
*Median (IQR)*	69 (58 – 76)	69 (59 – 76)	67 (56 – 77)	63 (51 – 77)	69 (66 – 78)
Female sex *n(%)*	242 (39)	204 (40)	22 (29)	17 (31)	5 (24)
Blood pressure (mmHg) *Mean (SD)*					
Systolic	139 (22)	139 (22)	139 (24)	139 (22)	140 (28)
Diastolic	77 (14)	77 (15)	78 (13)	80 (12)	73 (14)
Body mass index (kg/m2) *Median (IQR)*	27 (24 – 30)	27 (24 – 29)	28 (24 – 31)	27 (24 – 30)	30 (26 – 34)
Habitual alcohol consumption *n(%)*	217 (35)	179 (36)	23 (31)	15 (27)	8 (38)
Current smoker *n(%)*	171 (28)	139 (28)	22 (30)	17 (31)	5 (24)
Total cholesterol (mg/dl) *mean (SD)* b	198 (48)	199 (48)	198 (50)	204 (51)	180 (42)
High-density lipoprotein (mg/dl) *mean (SD)* c	51 (16)	52 (16)	49 (17)	50 (18)	47 (13)
Low-density lipoprotein (mg/dl) *mean (SD)* c	122 (41)	122 (41)	124 (43)	128 (43)	112 (40)
Triglyceride (mg/dl) *mean (SD)* d	139 (80)	136 (80)	152 (80)	152 (80)	152 (81)
History of: *n(%)*					
Hypertension	406 (65)	336 (66)	46 (61)	30 (55)	16 (76)
Diabetes mellitus	137 (22)	107 (21)	21 (28)	13 (24)	8 (38)
Peripheral artery disease	42 (7)	34 (7)	6 (8)	3 (6)	3 (14)
Coronary heart disease	99 (16)	80 (16)	16 (21)	10 (18)	6 (29)
Atrial fibrillation	132 (21)	106 (21)	18 (24)	11 (20)	7 (33)
Estimated GFR (ml/min) *mean (SD)*	77 (21)	77 (21)	79 (22)	83 (21)	70 (22)
NIHSS *median (IQR)*	2 (1 – 4)	2 (1 – 4)	3 (1 – 5)	2 (1 – 5)	3 (2 – 5)
NIHSS 0–4 *n (%)*	470 (76)	386 (76)	54 (71)	40 (73)	14 (67)
NIHSS 5–15 *n (%)*	151 (24)	121 (24)	22 (29)	15 (27)	7 (33)
TOAST *n (%)*					
Arterial atherosclerosis	167 (27)	128 (25)	25 (33)	17 (31)	8 (38)
Cardioembolic	145 (23)	121 (24)	18 (24)	12 (22)	6 (29)
Small vessel disease	96 (15)	87 (17)	6 (8)	4 (7)	2 (10)
Other	22 (4)	15 (3)	2 (3)	2 (4)	0
Undetermined etiology	191 (31)	156 (31)	25 (33)	20 (36)	5 (24)
Presence of chronic infarct lesions in MRIe,f					
* N (%)*	114 (26)	94 (27)	10 (23)	7 (21)	3 (28)
MR-DWI lesion volume in ml e,g *median (IQR)*	1.04 (0.35 – 4.49)	0.94 (0.30 – 3.71)	1.67 (0.41 – 6.07)	1.52 (0.37 – 4.32)	2.13 (0.73 – 14.55)
Years of school *n (%)*					
≤ 10	421 (68)	345 (72)	51 (68)	34 (63)	17 (81)
> 10	171 (28)	136 (28)	24 (32)	20 (37)	4 (19)
MMSE *median (IQR)*	28 (26 – 30)	28 (26 – 30)	29 (27 – 30)	29 (27.5 – 30)	27 (24 – 29)
Cognitive impairment (MMSE ≤ 26) *n(%)*	169 (28)	144 (29)	16 (22)	8 (15)	8 (40)

*SD* standard deviation, *IQR* interquartile range between the 25th and 75th percentile, *MI* myocardial infarction, *PAD* peripheral artery disease, *CHD* coronary heart disease, *BMI* body mass index, *GFR* glomerular filtration rate calculated using the chronic kidney disease epidemiology collaboration (CKD-EPI) formula, *HDL* high-density lipoprotein, *LDL* low-density lipoprotein, *NIHSS* National Institutes of Health Stroke Scale, *TOAST* stroke etiology according to Trial of Org 10,172 in Acute Stoke Treatment,* mRS* modified Rankin Scale, *MMSE* mini mental state examination; a antibody measurements were missing for 38 participants; missing values were < 10% in all characteristics except for b ‘total cholesterol’ missing: *n* = 57, c ‘HDL’ and ‘LDL’ missing: *n* = 38, d ‘Triglycerides’ missing: *n* = 49; eMRIs obtained retrospectively with different MRIs and protocols, f ‘presence of chronic infarct lesions in MRI’ missing: *n* = 203; gMR-DWI, magnet resonance diffusion-weighted imaging. Due to rounding, values might not add to 100%

### Follow-up data

**Fig. 2:**
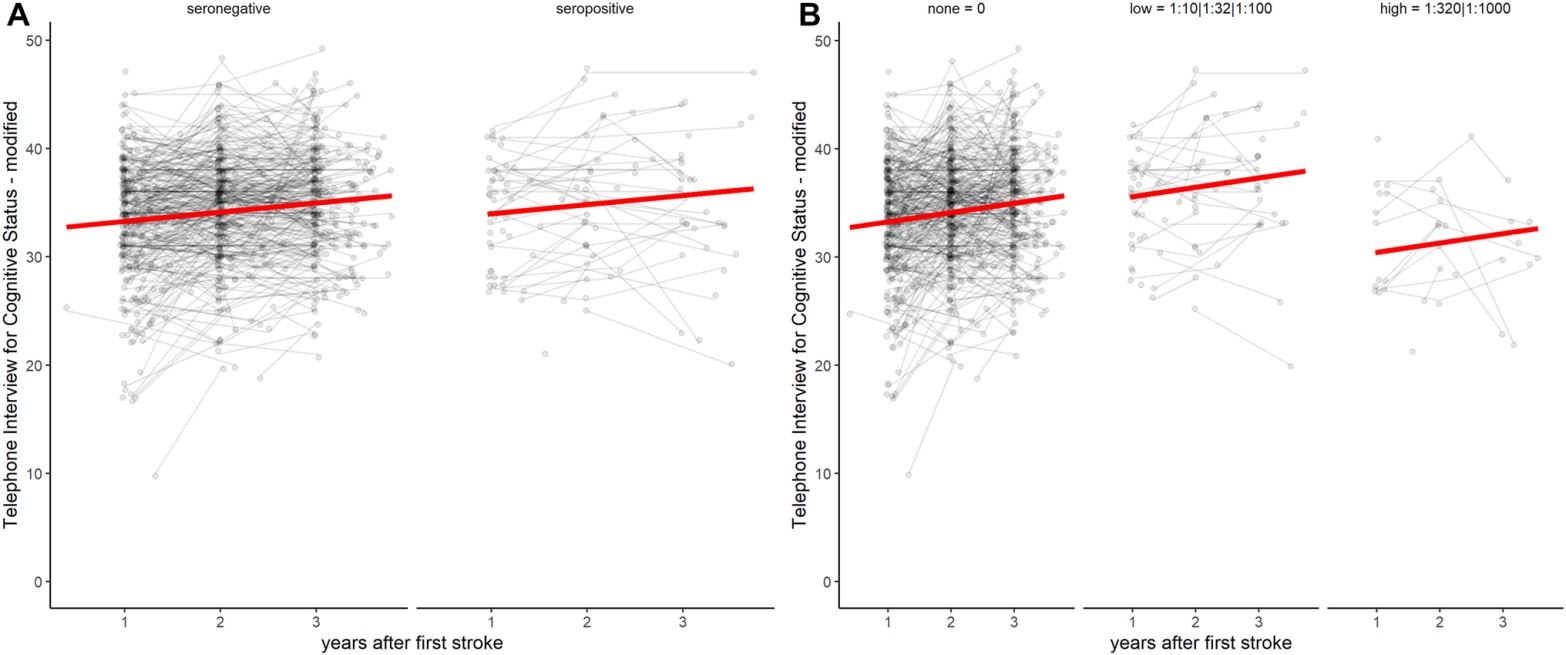
Anti-NMDA-receptor GluN1 antibody seropositive and seronegative patients and cognitive function (TICS-m Scores) after the first stroke. Cognitive function sum scores assessed with the Telephone Interview for Cognitive Status-modified (TICS-m) for A, anti-NMDA-receptor GluN1 antibody (NMDAR1-abs) seropositive and NMDAR1-abs seronegative patients and B, for NMDAR1-abs seropositive patients with low serum titers (titers of 1:10–1:100) and high serum titers (titers of 1:320 and 1:1000). Gray dots represent observed values, combined by respective subject. Red lines represent fitted lines over time from weighted linear mixed models

Annual TICS-m scores are visualized in Fig. 2, stratified by NMDAR1-abs subgroups. We did not observe a clinically relevant effect of NMDAR1-abs seropositivity compared to seronegativity on TICS-m regarded over three annual TICS-m assessments in the crude model (βcrude = 0.69 [95%CI  – 0.84 to 2.22]) and in propensity score-adjusted analyses (βadjusted = 0.65 [95%CI  – 1.00 to 2.30]). In patients with low titers of NMDAR1-abs, TICS-m scores over time were higher compared to seronegative patients in the crude (βcrude = 2.33 [95%CI 0.76–3.91]) and in the propensity score adjusted analysis (βadjusted = 2.47 [95%CI 0.75–4.19]). In patients with high NMDAR1-abs titers, TICS-m scores over time were lower compared to seronegative patients in the crude (βcrude =  – 2.82 [95%CI  – 4.90 to  – 0.74]) and in the adjusted analysis (βadjusted =  – 2.96 [95%CI  – 5.13 to  – 0.80]). For an overview see also Table 2

**Table 2:** Anti-NMDA-receptor antibody seropositivity and cognitive function over time after stroke

Serostatus	Crudea		Adjustedb	
	β	95% CI	β	95% CI
Seronegative	(ref.)	–	(ref.)	–
Seropositive	0.69	– 0.84 to 2.23	0.65	– 1.00 to 2.30
Titers ≤ 1:100	2.33	0.76 to 3.91	2.47	0.75 to 4.19
titers > 1:100	– 2.82	– 4.90 to – 0.74	– 2.96	– 5.13 to – 0.80

Serostatus, anti-NMDAR antibody seroprevalence. β, effect size (points on the Telephone Interview for Cognitive Status-modified [TICS-m]) in relation to the reference group. 95% CI, 95% confidence interval. *ref.*, reference category. aCrude, unadjusted analysis. bAdjusted, analysis adjusted for a propensity score built from age, sex, years of school education, smoking, alcohol consumption and the Trial of Org 10,172 in Acute Stoke Treatment (TOAST) classification for stroke etiology using logistic regression (binary outcome: seropositive and seronegative) and an ordinal logistic regression (titer level subgroups titers > 1:10 ≤ 1:100 and titers > 1:100) categories

### Results from sensitivity analyses

Characteristics of patients with at least one TICS-m assessment contrasted to patients with no TICS-m assessment at all are shown in Supplemental Table II. No clinically relevant differences could be observed, except that the prevalence of seropositivity was lower in patients with no TICS-m assessment at all (7% vs. 14%). After excluding TICS-m observations from 38 patients, who took antidementia (*n* = 7) and/or antidepressive drugs (*n* = 36) at baseline, the effect sizes from propensity score-adjusted IPW GLMs were not majorly different from those in the main analysis comparing seropositive with seronegative patients (adjusted β = 1.07; 95%CI  – 0.49 to 2.62) and seropositive subgroups with seronegative patients (adjusted βlow titer = 2.44; 95%CI 0.64–4.25; adjusted βhigh titer =  – 2.53; 95%CI  – 4.99 to −0.07, see Supplemental Table 3). In an additional analysis, the effects for titer subgroups of NMDAR1-abs seropositivity on TICS-m over time were attenuated after the exclusion of observations from depressed patients in a best-case and worst-case scenario. We provide full data in Supplemental Table 4, although this data should be interpreted with caution due to low numbers.

## Discussion

In our study of mild-to-moderately affected first-ever ischemic stroke patients, NMDAR1-abs seropositivity was not associated with cognitive function regarded over 3 years after the first ischemic stroke. However, we observed a dichotomy between patients with low and high NMDAR-abs titers: patients with low titers (≥ 1:10 – ≤ 1:100) performed better and patients with high titers (> 1:100) performed worse on annual cognitive testing compared to seronegative patients. Our data remains inconclusive whether the observed effects of NMDAR1-abs serostatus on cognitive function is mediated by depression.

A high serum prevalence of mainly the IgM and IgA NMDAR1-abs in various healthy and disease population, including stroke [4, 5, 9, 23, 24], questions a pathological significance of these isotypes on their own. However, in other non-stroke patient cohorts seropositivity was associated with cognitive impairment in melanoma patients, and iserum IgA was found to be associated with different types of slowly progressive cognitive impairment [4, 6, 7]. In stroke pathology, NMDAR1-abs seropositivity was previously linked to beneficial effects due to the presumed effects of NMDAR1-abs on NMDA receptor-mediated excitotoxicity [8, 9, 25]. Our group, however, observed increased vascular risk and mortality in seropositive patients and worse functional outcome in patients with high NMDAR1-abs titers [15]. Despite methodological limitations (i.e., different machines and protocols, high amount of missing data) our MRI parameters do not support a hypothesis of beneficial effects of NMDAR1-abs seropositivity on infarct lesion volumes: MR-DWI lesion volumes were larger in seropositive patients, particularly in those with high titers (see Table 1.) compared to seronegative patients. Taken together, our findings challenge hypotheses of beneficial effects of these antibodies in stroke pathology.

Although we did not measure serostatus before the acute event, there are strong arguments supporting the preexistence of serum NMDAR1-abs before the stroke: first, it takes a minimum of 4 days to generate any antibody after antigen presentation,(26) thus titers as measured in our study are unlikely to be observed after such a short time if the antibodies were not preexisting (median day of blood sampling after stroke = 4, IQR: 3–5). This is further supported by NMDAR1-abs measurements of another stroke cohort (STRAWINSKI, Identifier NCT01264549), in which we measured the serostatus within 36 h after the stroke and observed a similar seroprevalence of IgA and IgM antibodies (data not published) as in the PROSCIS-B study. Second, there was no relationship between NMDAR1-abs titers and day of blood sampling after the stroke (median day of blood sampling in seropositive patients with low titers = 4, [IQR = 3–6]; and in patients with high titers = 4, [ IQR = 3-5]); if antibodies were formed as a consequence of the stroke, we would have expected titers to increase over time. Last, a body of research shows a similar NMDAR1-abs serum prevalence in other disease cohorts as well as in healthy subjects.[5, 6, 10, 27] We consider that preexisting serum NMDAR1-abs enter brain parenchyma as a consequence of stroke where they may downregulate NMDA receptors and hamper NMDAR function and functional recovery of the damaged tissue [23].

A recent study including 114 ischemic stroke patients found associations of NMDAR1-abs seropositivity with neuropsychiatric outcomes including cognition [26]. Our result show diverging effects of low and high NMDAR1-abs titers on cognitive function after stroke.

A paradox has been described regarding the functional properties of the NMDA receptor previously: only excessive NMDA-receptor activation leads to detrimental effects, for example, in ischemic brain damage, whereby physiological NMDA-receptor activation is important for neuroplasticity and regeneration [3, 25, 27]. We put our findings into this context: whilst low titers of NMDAR1-abs may not be sufficient to hamper physiological NMDA-receptor function after stroke, high NMDAR1-abs titers may impair physiological NMDA-receptor function and subsequently reorganization of the damaged brain. This could result in decreased cognitive performance as observed in this study. Therefore, this non-linear dose–response relationship fits into the biology of the NMDA receptor. A reasonable cutoff value for NMDAR1-abs seropositivity as a potential risk factor for unfavorable outcome after stroke is yet to be established.

In our study, NMDAR1-abs measurements were done in a highly qualified laboratory with a standardized commercially available fixed CBA. There is some ongoing controversy regarding the superiority of fixed CBA versus CBA using living cells (live CBA), for the detection of serum NMDAR1-abs. While some reports suggested an increased sensitivity for live CBAs compared to fixed CBAs, based on less epitope damage from fixation,[30–33] others found no differences.[34] Obvious advantages of the fixed CBA are the easy handling, storage, comparability outside of specialized laboratories and the routine applicability.[33] Importantly, even a minimally improved sensitivity is highly unlikely to have resulted in different outcomes between patient groups in our study. Similarly, a controversy exists regarding whether the different immunoglobulin classes (i.e., IgA and IgM) affect neuronal NMDARs in a clinically relevant manner,[8, 23, 24] awaiting experimental confirmation using patient-derived monoclonal IgA/IgM autoantibodies. Our data complements a number of previous studies showing cognitive deficits in patients with serum NMDAR1-abs of primarily the IgA and IgM isotypes,[5, 7, 8, 28, 35] supporting the pathogenic effects of these antibodies beyond the acute encephalitis spectrum. The clinical phenotype and pathobiology of NMDAR1-abs seropositivity in stroke and other diseases demands further characterization, which is important to guide diagnostic approaches. In a tissue-based assay using primate cerebellum, only five patient sera showed staining in either the molecular or nuclear cell layer (four with IgA antibodies and three with IgM with low titers except for one case with IgA titers of 1:320).

We consider the observed effects as clinically meaningful, supported by effect sizes representing half an SD of overall TICS-m results in the PROSCIS–B cohort (SD of TICS-m at year 1 after stroke = 5.4 points). In one of our sensitivity analyses, we excluded TICS-m observations of patients who we considered depressed, in a best-case (all patients with missing information were considered not depressed, thus contributing their TICS-m observations to the analysis) and a worst-case scenario (all patients with missing information were considered to be depressed, thus their TICS-m observations were excluded from the analysis), to exclude that potential effects may be mediated through depression. Our observed effects were attenuated in this significantly reduced sample size. Maybe a significant proportion of our patients with decreased cognitive function suffer comorbid depression, or else a significant proportion of patients are depressed only and present with pseudodementia due to depression, thus scoring lower in cognitive testing.

## Limitations

A learning effect in cognitive test scores may bias our results toward the null [28]. Furthermore, we were not able to conduct a detailed neuropsychological testing, which could discriminate cognitive domains and even minor disabilities. To reduce internal heterogeneity, we excluded six patients with a baseline NIHSS ≥ 16. However, differences in cognitive function may be more pronounced in severely affected stroke patients and our study results cannot be generalized to patients with severe stroke events. Repeated antibody measurements, acutely and during follow-up, would have allowed to assess titer-level stability and possible immunoglobulin class switches, which however were not available for this study. Selection bias may be present, as we recorded missing data in our outcome variable. To address this concern, we used a time-specific weighted model under the assumption that missing observations from subjects who had no further follow-up were not randomly missing (e.g., from patients who died). Titer level cutoff to define subgroups in this cohort was prespecified before the analyses, however, at our discretion. To confirm the observed results, further prospective studies with similar patient groups are welcome.

## Conclusion

In our study, cognitive outcome of ischemic stroke patients with any NMDAR1-abs seropositivity, primarily with IgA and IgM, was similar to that of seronegative patients over 3 years. Patients with high NMDAR1-abs titers (> 1:100) had worse cognitive outcome compared to seronegative patients. Diverging associations of patients with different titer levels reflect either a complex regulatory system of the NMDA receptor or a similarly complex immunobiology. The observed effects demand further confirmation.

## Supplementary Information

Below is the link to the electronic supplementary material.

Supplementary file1 (DOCX 472 KB)

## Declarations

### Statement of responsibility

P.S. Sperber, T.G. Liman and B. Siegerink take full responsibility for the clinical data, the analyses and interpretation, and the conduct of the research, have full access to all data, and have the right to publish any and all data.

### Statistical analyses

Conducted by P.S. Sperber, reviewed by P. Gebert.
