# Supplementary material for: Serum anti-NMDA-receptor antibodies and cognitive function after ischemic stroke (PROSCIS-B)
Source: J Neurol. 2022 Jun 19;269(10):5521–30. doi: 10.1007/s00415-022-11203-x (PMC9468072; doi:10.1007/s00415-022-11203-x)
Supplement: Supplementary file 1 — Supplementary file1 (DOCX 472 KB) [file 415_2022_11203_MOESM1_ESM.docx]

**SUPPLEMENTAL MATERIAL**

**Serum anti-NMDA-receptor antibodies and cognitive function after ischemic stroke (PROSCIS-B)**

Pia S. Sperber^1,2,3,4^, Pimrapat Gebert^5,6^, Leonie H.A. Broersen^1^, Shufan Huo^1,3,7^, Sophie K. Piper^5,6^, Bianca Teegen^8^, Peter U. Heuschmann^9,10^, Harald Prüss^3,7^, Matthias Endres^1,2,3,4,7^, Thomas G. Liman^1,7^*, Bob Siegerink^1,11^*

*equal contribution

^1^Charité – Universitätsmedizin Berlin, Center for Stroke Research Berlin (CSB), Berlin, Germany

^2^German Centre for Cardiovascular Research DZHK, partner site Berlin, Germany

^3^German Center for Neurodegenerative Disease DZNE, partner site Berlin, Germany.

^4^Charité – Universitätsmedizin Berlin, NeuroCure Clinical Research Center (NCRC), Berlin, Germany.

^5^Charité – Universitätsmedizin Berlin, Institute of Biometry and Clinical Epidemiology, Berlin, Germany

^6^Berlin Institute of Health (BIH), Charité – Universitätsmedizin Berlin and Max Delbrück Center for Molecular Medicine in the Helmholtz Association, Berlin, Germany

^7^Charité – Universitätsmedizin Berlin, Department of Neurology with Experimental Neurology, Berlin, Germany.

^8^Institute of Experimental Immunology, EUROIMMUN AG, Luebeck, Germany.

^9^University of Würzburg, Institute of Clinical Epidemiology and Biometry, Würzburg, Germany.

^10^University Hospital Würzburg, Clinical Trial Center Würzburg, Würzburg, Germany.

^11^Department of Clinical Epidemiology, Leiden University Medical Center, Leiden, the Netherlands.

Supplemental Methods 1. **Question items of the Telephone Interview for Cognitive Status – modified (TICS-m)**

1. name, age, date, season and phone number (9 points), 2. counting backwards (2 points), 3. a learning exercise of 10 words and a delayed recall of these words (10 points each), 4. subtractions (5 points), 5. responsive naming (4 points) 6. repetition (2 points), 7. current president and chancellor (4 points), 8. finger tapping (worth 2 points), and 9. word opposites (2 points).

Supplemental Methods 2. **Inverse probability weighting in a generalized linear model.**

We recorded missing observations in our outcome variable (TICS-m), therefore we used an inverse probability weighted generalized linear model to prevent bias due to non-random missingness.(1) We first classified missing TICS-m observations into two types of missing information: non-monotonic missing values (also called intermitting missing observations: e.g. a missing TICS-m score from a subject two years after the stroke, when the respective subject provided a score at one and three years after the stroke) and monotonic-missing values (i.e. no further TICS-m score could be obtained). Non-monotonic missings were considered missing completely at random (MCAR) because we assumed the missing pattern to be independent of cognitive outcome or other variables.(1) MCAR missings do not lead to biased results in a GLM and were therefore disregarded. Monotonic missingness (i.e. missing after truncation, including those missing observations due to death), however, was considered not at random (MNAR). Those MNAR missings were handled in the model with time-specific weights on available observations. The weights were set on continuation in the study period. We included time in years on a continuous scale as a time variable and the patient identifier (patient ID) to indicate dependencies of the outcomes (autocorrelation) due to repeated TICS-m measurements within one subject. We applied this approach with the xtrccipw built-in STATA command, which has been described previously in full detail.(2)

Supplemental Methods 3: **Confounder selection and strategy**

Confounding variables were defined as those variables that would have a possible impact on both, NMDAR1-abs serostatus and cognitive function after stroke and were selected by a causal diagram (concept of conditional independencies)(3) for which we took the variables age (continuous), sex (binary), education (≤ 10 years of school; >10 years of school corresponding to low-to-middle level and high level education according to the German schooling system), socio economic status (unobserved), body mass index (BMI, kg/m^2^), history of arterial hypertension (yes/no), myocardial infarction (yes/no), peripheral artery disease (yes/no), diabetes mellitus (yes/no), atrial fibrillation (yes/no), current smoking (yes/no), habitual alcohol consumption (yes/no), systemic inflammation (unobserved) and the Trial of Org 10172 in Acute Stroke Treatment (TOAST)-criteria (categories: 1.large-artery atherosclerosis, vs. 2.cardioembolism /3.small vessel occlusion /4.other cause / 5.unknown etiology) into consideration, as shown in Supplemental Figure 1. All pre-stroke comorbidities that would potentially confound an effect of NMDAR1-abs on cognitive function after stroke (i.e. arterial hypertension, myocardial infarction, diabetes mellitus, peripheral artery disease and atrial fibrillation) in our data are represented by pathologies leading to stroke etiologies classified by the TOAST criteria, and we therefore decided to include only the TOAST criteria variable instead of all the comorbidities singularly, to avoid overadjustment. We ultimately used age, sex, education in 2 categories, current smoking, habitual alcohol consumption, and the TOAST-criteria to calculate a propensity score for confounder adjustment in our analyses.

Supplemental Figure I. **Causal diagram: assumptions of variables conditional independencies**


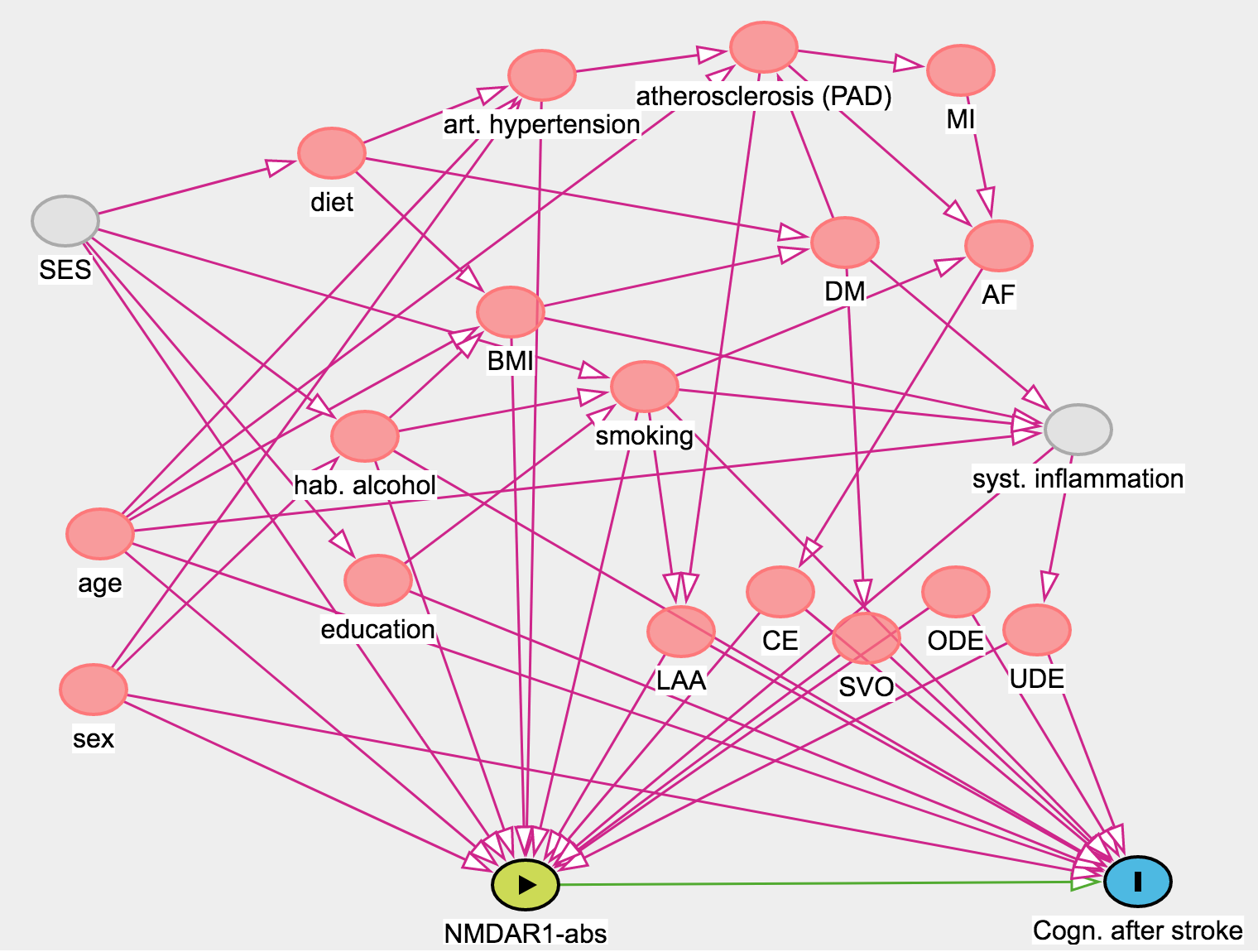


Abbreviations: SES, socio economic status; hab. alcohol, habitual alcohol consumption; BMI, body mass index, PAD, peripheral artery disease; MI, myocardial infarction; AF, atrial fibrillation; LAA, large-artery atherosclerosis; CE, cardioembolism; SVO, small vessel occlusion; ODE, other setermined stroke etiology; UDE, undetermined stroke etiology; NMDAR1-abs, anti-NMDA-receptor NR1 antibody serostatus; Cogn. after stroke, cognitive outcome after stroke; Conditional independencies are visualized using directed acyclic graphs (DAG) by dagitty (http://www.dagitty.net/dags.html) in which each connection represents a potential relationship (i.e. a possible relationship, which must not be true) between the variables (nodes). The arrows indicate how variables are assumed to relate to each other. If the arrow is pointing towards the one variable it means that the variable the arrow stems from potentially impacts the other (e.g. age is assumed to impact arterial hypertension, atherosclerosis, BMI, systemic inflammation, seroprevalence of NMDAR1-abs and cognitive function after stroke. The research question is represented with a green arrow: the impact of NMDAR1-abs on cognitive function after stroke. This causal model rendered that age, sex, education, hab. alcohol, smoking CE, LAA, ODE, SVO, UDE (representing the Trial of Org 10172 in Acute Stroke Treatment (TOAST)-criteria) are a sufficient set of variables for adjustment to estimate the effect of NMDAR1-abs on cognitive function after stroke.

Supplemental Methods 4: **Drugs taken at baseline that led to exclusion from sensitivity analyses.**

Anti-dementia drugs: Anatomic Therapeutic Chemical [ATC] codes N06DA [Anticholinesterases], N06DX [Other anti-dementia drugs]

Antidepressants: ATC codes N06AA [Non-selective monoamine reuptake inhibitors], N06AB [Selective serotonin reuptake inhibitors], N06AF [Monoamine oxidase inhibitors, non-selective], N06AG [Monoamine oxidase A inhibitors], N06X [other antidepressants].

Supplemental Methods 5: **Definitions of depression at year one, two and three years after stroke and the best-case as well as the worst-case scenario for exclusion of observations for sensitivity analyses**

We used the Center for Epidemiologic Studies Depression Scale (CES-D), a screening instrument for depression, which was assessed at each follow up time point and we considered values ≥16 as presence of depression.(4) In the CES-D, 20 single likert-type questions brings up to 3 points, which then in turn add up to a maximum of 60 possible points. TICS-m scores that were obtained while a patient had simultaneously a CES-D of ≥16 were subsequently excluded from analyses. We also observed missing items in CES-D and therefore calculated effects for a best-case scenario (BC) in which missing items of the CES-D questionnaire were replaced with the minimal score (0 points) for each question, resulting in the least amount of depressed patients and a worst-case scenario (WC), in which missing items of the CES-D questionnaire were replaced with the maximal score (3 points), resulting in the greatest amount of excluded TICS-m observations.

Supplemental Table I: Coefficients of difference between N-Methyl-D-Aspartate-Receptor GluN1 antibody seropositive and seronegative patients, and respective seropositive subgroups

|  | **seropos. vs. seroneg.**  **patients** | **low titer group vs. seroneg. patients** | **high titer group vs. seroneg. patients** |
| --- | --- | --- | --- |
| **age (years**) *diff. in means (95% CI)* | -1 (-4 – 2) | -3 (-6 – 1) | 4 (-2 – 10) |
| **female sex** *diff. in percentage points (95% CI)* | -11 (-22 – <0) | -9 (-22 – <0) | -16 (-35 – 3) |
| **blood pressure (mmHg)** *diff. in means (95% CI)*  **systolic**  **diastolic** | <0 (-5 – 6)  1 (-2 – 5) | <0 (-6 – 6)  3 (-1 – 7) | 1 (-8 – 11)  -4 (-10 – 2) |
| **body mass index (kg/m^2^)** *diff. in means (95% CI)* | >0 (<0 – 2) | <0 (-2 – 1) | 2 (>0 – 5) |
| **hab. alcohol consumpt.** *diff. in percentage points (95% CI)* | -5 (-16 – 6) | -8 (-21 – 4) | 4 (-18 – 25) |
| **current smoker** *diff. in percentage points (95% CI)* | 2 (-9 – 14) | 5 (-8 – 18) | -4 (-22 – 14) |
| **total cholesterol (mg/dl)** *diff. in means (95% CI)* | -1 (-13 – 11) | 5 (-8 – 19) | -19 (-41 – 6) |
| **high density lipoprotein** **(mg/dl)** *diff. in means (95% CI)* | -3 (-6 – 1) | -2 (-6 – 3) | -5 (-12 – 2) |
| **low density lipoprotein (mg/dl)** *diff. in means (95% CI)* | 2 (-8 –12) | 6 (-5 – 18) | -10 (28 – 8) |
| **triglyceride (mg/dl)** *diff. in means (95% CI)* | 16 (-4 – 35) | 15 (-8 – 38) | 16 (-20 –52) |
| **history of:**  *diff. in percentage points* *(95% CI)*  **hypertension**  **diabetes mellitus**  **peripheral artery disease**  **coronary heart disease**  **atrial fibrillation** | -6 (-17 – 6)  7 (-4 – 17)  1 (-5 – 8)  5 (-4 –15)  3 (-7 – 13) | -11 (-26 – 2)  3 (-9 – 14)  -1 (-8 – 5)  2 (-8 – 13)  <0 (-12 – 10) | 9 (-9 – 29)  17 (-4 – 39)  8 (-8 – 23)  13 (-7 – 33)  12 (-8 – 33) |
| **estimated GFR (ml/min)** *diff. in means (95% CI)* | 3 (-2 – 8) | 6 (>0 – 12) | -6 (-15 – 3) |
| **NIHSS** *diff. in ranks (95% CI)* | >0 (<0 – 1) | <0 (-1 – >0) | >0 (<0 – 1) |
| **NIHSS 0 – 4** *diff. in percentage points (95% CI)* | -5 (-15 – 6) | -3 (-16 – 9) | -9 (-30 – 11) |
| **NIHSS 5 – 15** *diff. in percentage points (95% CI)* | 5 (-6 – 15) | 3 (-9 – 16) | 9 (-11 – 30) |
| **TOAST** *diff. in percentage points (95% CI)*  **arterial atherosclerosis**  c**ardioembolic**  **small vessel disease**  **other**  **undetermined etiology** | 8 (-4 – 18)  <0 (-10 – 10)  -9 (-16 – -2)  <0 (-4 – 4)  2 (-9 – 13) | 6 (-7 – 19)  -2 (-13 – 10)  -10 (-18 – -2)  >0 (-4 – 6)  6 (-8 – 19) | 13 (-9 – 34)  5 (-15 – 24)  -8 (-21 – 5)  -3 (-4 – -1)  -7 (-25 – 12) |
| **Presence of chronic infarct lesions in MRI**^e,f^  *diff. in percentage points (95% CI)* | -4 (-17 – 9) | -6 (-21 – 8) | >0 (-3 – 3) |
| **MR-DWI lesion volume in ml** ^e,g^  *diff. in log. means (95% CI)* | 0.48 (-0.09 – 1.04) | 0.26 (-0.38 – 0.91) | 1.13 (0.04 – 2.22) |
| **years of school** *diff. in percentage points (95% CI)* **≤10**  **>10** | -4 (-15 – 7)  4 (-7 – 15) | -9 (-23 – 5)  9 (-5 – 23) | 9 (-8 – 27)  -9 (-27 – 8) |
| **MMSE** *diff. in means (95% CI)* | 1 (>0 – 1) | 1 (<0 – 2) | -1 (-2 –1) |
| **Cognitive impairment (MMSE≤26)** *diff. in percentage points (95% CI)* | -6 (-17 – 4) | -13 (-24 – -2) | 11 (-11 – 34) |

Seropos. N-Methyl-D-Aspartate-Receptor GluN1 antibodies (NMDAR1-abs) seropositive patients; seroneg. NMDAR1-abs seronegative patients; low titer group, patients with low titers (1:10-1:100) of NMDAR1-abs; high titer group, patients with high titers (>1:100) of NMDAR1-abs; diff, difference; IQR, inter quartile range between the 25^th^ and 75^th^ percentile; BMI, Body Mass Index; MI, myocardial infarction; PAD, peripheral artery disease; CHD, coronary heart disease; GFR, glomerular filtration rate calculated using the Chronic Kidney Disease Epidemiology Collaboration (CKD-EPI) formula; HDL, high density lipoprotein; LDL, low density lipoprotein; NIHSS, National Institutes of Health Stroke Scale; TOAST, stroke etiology according to Trial of Org 10172 in Acute Stoke Treatment; mRS, modified Rankin Scale; MMSE, Mini Mental State Examination ^a^38 participants were missing antibody measurements; Missing values were < 10% in all characteristics except for ^b^‘total cholesterol’ missing: n = 57, ^c^‘HDL’ and ‘LDL’ missing: n = 38 , ^d^‘Triglycerides’ missing: n = 49; ^e^MRIs obtained retrospectively with different MRIs and protocols, ^f^‘presence of chronic infarct lesions in MRI’ missing: n = 203; ^g^MR-DWI, magnet resonance defusion weighted imaging; Due to rounding values might not add up to values provided in Table 1.

Supplemental Table II. **Baseline Characteristics Table of PROSCIS – B Participants** **stratified to patients with at least one TICS-m measurement and patients missing all TICS measurements**

|  | **At least one TICS-m** | **Missing TICS-m completely** |
| --- | --- | --- |
| **Participants** * *n (%)* | 491 (79) | 130 (21) |
| **Anti-NMDAR GluN1 antibody seropositive** *n (%)* | 67 (14)* | 9 (7)* |
| **Age (years**) *mean (SD)* | 67 (13) | 68 (13) |
| **Age (years**) *median (IQR)* | 69 (58 – 76) | 68.5 (59 – 78) |
| **Female sex** *n (%)* | 191 (39) | 51 (39) |
| **Blood pressure (mmHg)** *mean (SD)* |  |  |
| Systolic | 139 (22) | 142 (23) |
| Diastolic | 77 (14) | 77 (16) |
| **History of hypertension** *n (%)* | 315 (64) | 91 (70) |
| **BMI (kg/m^2^)** *median (IQR)* | 28 (24 – 30) | 27 (25 – 30) |
| **Habitual alcohol consumption** *n (%)* | 175 (36) | 42 (32) |
| **Current smokers** *n (%)* | 126 (26) | 45 (35) |
| **Cholesterol (mg/dl)** *mean (SD)* |  |  |
| Total‡ | 200 (48) | 192 (48) |
| HDL§ | 52 (16) | 49 (14) |
| LDL§ | 124 (41) | 116 (39) |
| **Triglycerides (mg/dl)** \| \| *mean (SD)* | 138 (78) | 142 (89) |
| **History of diabetes mellitus** *n (%)* | 106 (22) | 31 (24) |
| **History of arterial disease** *n (%)* |  |  |
| PAD | 32 (7) | 10 (8) |
| CHD | 78 (16) | 21 (16) |
| **eGFR** *mean (SD)* | 77 (21) | 77 (22) |
| **History of atrial fibrillation** *n (%)* | 108 (22) | 24 (18) |
| **NIHSS** *median (IQR)* | 2 (1 – 4) | 3 (1 – 5) |
| 0 – 4 | 374 (76) | 96 (74) |
| 5 – 16 | 117 (24) | 34 (26) |
| **TOAST** *n (%)* |  |  |
| Large-artery atherosclerosis | 136 (28) | 31 (24) |
| Cardioembolic | 114 (23) | 31 (24) |
| Small-vessel occlusion | 73 (15) | 23 (18) |
| Stroke of other determined etiology | 21 (4) | 1 (1) |
| Stroke of undetermined etiology | 147 (30) | 44 (34) |
| **mRS at baseline** *median (IQR)* | 2 (1 – 3) | 2 (1 – 3) |
| **Years of school education** *n (%)* |  |  |
| ≤ 10 years | 329 (67) | 92 (71) |
| > 10 years | 145 (30) | 26 (20) |
| **MMSE** *median (IQR)* | 29 (26 – 30) | 27 (25 – 29) |

SD, Standard deviation; IQR, inter quartile range between the 25^th^ and 75^th^ percentile; MI, myocardial infarction; PAD, peripheral artery disease; CHD, coronary heart disease; BMI, Body Mass Index; GFR, glomerular filtration rate calculated using the Chronic Kidney Disease Epidemiology Collaboration (CKD-EPI) formula; HDL, high density lipoprotein; LDL, low density lipoprotein; NIHSS, National Institutes of Health Stroke Scale; TOAST, stroke etiology according to Trial of Org 10172 in Acute Stoke Treatment; mRS, modified Rankin Scale; MMSE, Mini Mental State Examination *38 participants were missing antibody measurements; Missing values were < 10% in all characteristics except for ‡‘total cholesterol’ missing: n = 57, §‘HDL’ and ‘LDL’ missing: n = 38 , | |‘Triglycerides’ missing: n = 49; Due to rounding values might not add to 100%.

Supplemental Table III. **Anti-NMDA-Receptor Antibody Seropositivity and Cognitive Function Over Time after Stroke** **after excluding patients with anti-dementia drugs and anti-depressants before stroke**

| **Serostatus** | **Unadjusted** | | **Adjusted** | |
| --- | --- | --- | --- | --- |
|  | **β** | 95% CI | **β** | 95% CI |
| seronegative | **0** | (ref.) | **0** | (ref.) |
| seropositive | **1.07** | -0.49 to 2.62 | **1.04** | -0.69 to 2.77 |
| titers ≤ 1:100 | **2.30** | 0.69 to 3.92 | **2.44** | 0.64 to 4.25 |
| titers > 1:100 | **-2.37** | -4.74 to -0.01 | **-2.53** | -4.99 to -0.07 |

Serostatus, anti-NMDAR antibody seroprevalence. β, effect size (points on the Telephone Interview for Cognitive Status-modified [TICS-m]) in relation to reference group. 95% CI, 95% confidence interval. ref., reference category. Grey shaded values are pooled estimates after multiple imputation of missing values. ^a^Adjusted, analysis adjusted for a propensity score built from age, sex, years of school education, smoking, alcohol consumption and the Trial of Org 10172 in Acute Stoke Treatment (TOAST) classification for stroke etiology using logistic regression (binary outcome: seropositive and seronegative) and an ordinal logistic regression (titer level subgroups titers >1:10 ≤ 1:100 and titers > 1:100).

Supplemental Table IV. **Anti-NMDA-receptor antibody GluN1 Seropositivity and Cognitive Function over Time after Stroke** **after Exclusion of Observations obtained while the patient was depressed: Best- and Worst-Case Scenario**

| **serostatus** | BC or WC | **Unadjusted** | | **Adjusted**^a^ | |
| --- | --- | --- | --- | --- | --- |
|  | Observations Excluded (n [%]) | **β** | 95% CI | **β** | 95% CI |
| seronegative | BC (258 [17%]) | **1** | (ref.) | **1** | (ref.) |
|  | WC (768 [50%]) |  |  |  |  |
| seropositive | BC (44 [19%]) | **1.00** | -0.52 to 2.53 | **1.22** | -0.38 to 2.82 |
|  | WC (124 [54%]) | **1.11** | -0.40 to 2.61 | **0.88** | -0.70 to 2.45 |
| titers ≤ 1:100 | BC (30 [18%]) | **2.09** | 0.34 to 3.84 | **2.45** | 0.63 to 4.27 |
|  | WC (86 [52%]) | **1.99** | 0.27 to 3.72 | **1.80** | -0.01 to 3.62 |
| titers > 1:100 | BC (14 [22%]) | **-1.81** | -4.55 to 0.93 | **-1.92** | -4.81 to 0.96 |
|  | WC (38 [60%]) | **-1.28** | -4.02 to 1.47 | **-1.42** | -4.28 to 1.44 |

Serostatus, anti-NMDAR antibody seroprevalence. β, effect size in relation to reference group. 95% CI, 95% confidence interval. ref., reference category. CES-D, Center for Epidemiological Studies Depression Scale. BC, best case scenario (missing items imputed with 0 points on CES-D score). WC, worst case scenario (missing items imputed with 3 points on CES-D score) ^a^Adjusted, analysis adjusted for a propensity score built from age, sex, years of school education, smoking, alcohol consumption and the Trial of Org 10172 in Acute Stoke Treatment (TOAST) classification for stroke etiology using logistic regression (binary outcome: seropositive and seronegative) and an ordinal logistic regression (titer level subgroups titers >1:10 ≤ 1:100 and titers > 1:100).

**References:**

1. Rubin DB. Inference and missing data. Biometrika. 1976;63(3):581–92.

2. Daza EJ, Hudgens MG, Herring AH. Estimating inverse-probability weights for longitudinal data with dropout or truncation: The xtrccipw command. Stata J. 2017;17(2):253–78.

3. Williams TC, Bach CC, Matthiesen NB, Henriksen TB, Gagliardi L. Directed acyclic graphs: a tool for causal studies in paediatrics. Pediatr Res [Internet]. 2018;84(4):487–93. Available from: http://dx.doi.org/10.1038/s41390-018-0071-3

4. Stein J, Luppa M, Mahnke J, Weyerer S, Schomerus G, Riedel-Heller SG. Depressionsscreening am Telefon mittels der Allgemeinen Depressionsskala (ADS): Ergebnisse einer bevölkerungsrepräsentativen Erhebung. Psychiatr Prax. 2014;41(3):135–41.
